# A high throughput messenger RNA differential display screen identifies discrete domains of gene expression and novel patterning processes along the developing neural tube

**DOI:** 10.1186/1471-213X-6-9

**Published:** 2006-02-24

**Authors:** David Chambers, Ivor Mason

**Affiliations:** 1 MRC Centre for Developmental Neurobiology, 4^*th *^Floor New Hunt's House, King's College London, Guy's Campus, London, SE1 1UL, UK; 2 Wellcome Trust Functional Genomics Development Initiative, MRC Centre for Developmental Neurobiology, 4^*th *^Floor New Hunt's House, King's College London, Guy's Campus, London, SE1 1UL, UK

## Abstract

**Background:**

During early development the vertebrate neural tube is broadly organized into the forebrain, midbrain, hindbrain and spinal cord regions. Each of these embryonic zones is patterned by a combination of genetic pathways and the influences of local signaling centres. However, it is clear that much remains to be learned about the complete set of molecular cues that are employed to establish the identity and intrinsic neuronal diversity of these territories. In order to address this, we performed a high-resolution messenger RNA differential display screen to identify molecules whose expression is regionally restricted along the anteroposterior (AP) neuraxis during early chick development, with particular focus on the midbrain and hindbrain vesicles.

**Results:**

This approach identified 44 different genes, with both known and unknown functions, whose transcription is differentially regulated along the AP axis. The identity and ontological classification of these genes is presented. The wide variety of functional classes of transcripts isolated in this screen reflects the diverse spectrum of known influences operating across these embryonic regions. Of these 44 genes, several have been selected for detailed *in situ *hybridization analysis to validate the screen and accurately define the expression domains. Many of the identified cDNAs showed no identity to the current databases of known or predicted genes or ESTs. Others represent genes whose embryonic expression has not been previously reported. Expression studies confirmed the predictions of the primary differential display data. Moreover, the nature of identified genes, not previously associated with regionalisation of the brain, identifies novel potential mechanisms in that process.

**Conclusion:**

This study provides an insight into some of the varied and novel molecular networks that operate during the regionalization of embryonic neural tissue and expands our knowledge of molecular repertoire used during development.

## Background

Even before neurulation begins, the neural plate is broadly patterned into domains with anterior and posterior molecular character. As development proceeds, the founding pattern is further refined upon until the morphologically characteristic structures of the forebrain, midbrain, hindbrain and spinal cord arise [1]. The molecular mechanism by which the acquisition of the anterior-posterior (AP) identity is achieved has been the subject of intense study. In recent years many fundamental patterning processes have been elucidated but relatively little is understood about the complete set of signalling influences or genetic networks used to achieve this neuronal diversity. However, it is known that the specification and subsequent patterning of the neural tube uses hierarchies of transcription factors working in combination with the activity of signalling centres located at discrete AP positions.

During gastrulation, *Otx2 *and *Gbx2 *are expressed in opposing domains in the neural plate, anteriorly and posteriorly respectively. Within the *Otx2 *positive domain the forebrain vesicle develops and there is good evidence for transcriptional regulation playing a deterministic patterning role of that region [[2-5]; reviewed by 1]. The interface between the zones of expression of *Otx2 *and *Gbx2 *uniquely determines the future position of the midbrain-hindbrain organizer {MHB, also referred to as isthmus), a structure intimately associated with correct patterning of the mid-hindbrain region [reviewed by [6-8]]. Subsequently, a series of transcription factors including *Pax2*, *Pax5*, *Pax8*, *En1*, *En2 *are expressed at the *Otx*2-*Gbx*2 boundary that are required for maintenance of the MHB organizer and specification of the midbrain territory. These transcription factors then participate together with the secreted signals Fgf8 and Wnt1 in a cross-regulatory network to maintain the MHB organizer and direct patterning of the midbrain and cerebellar structures [reviewed by [8]]. Multiple lines of evidence show that FGF8, which is expressed in the temporally appropriate manner at the MHB in all vertebrate classes [9-18], constitutes an important component of the isthmic-patterning signal. Introduction of ectopic FGF8 protein into the avian brain can respecify posterior forebrain to become midbrain and anterior midbrain to develop posterior midbrain characteristics [14,19,20,18]. Taken together, these data demonstrate the existence of a complex interplay of genetic regulation and local signalling that are required to specify and pattern the midbrain territory [reviewed by [21]].

The hindbrain becomes organized into a repeated series of cell lineage-restricted metametric units defined as rhombomeres (r) [[22], reviewed by [23]]. These compartments facilitate a coordination of AP and dorsoventral (DV) signals to regulate the pattern of the neuronal subtypes born within them such that each rhombomere can be considered to have a unique identity. Rhombomere 1 is notable in respect of its lack of branchial motor neurons and being the precursor region for the cerebellum. The anterior rhombic lip of r1 (a dorsolateral zone where the neuroepithelium abuts the roofplate) generate a large migratory cell population that forms the external germinal layer and later the internal granule cell layer of the cerebellum [24]. It is now well established that the hindbrain is patterned by the combinatorial action of the homeodomain-containing hox transcription factor code. Within the region that is initially defined as *Gbx*2 positive in the neural plate, the initial hox code in the hindbrain is first set up under the influence of AP signals such as retinoic acid (RA) [e.g. see [25]; reviewed by [26,27,23]]. Given the distribution of the hox transcripts and their ability to act in a co-operative fashion, it has been suggested that individual rhombomere identity is conferred by a combinatorial code of the hox proteins [28,26]. Functional evidence for the role of hox genes in the patterning of the hindbrain can be observed from interference studies on the *Hoxb*1 gene that is normally highly expressed in r4. Disruption of the *Hoxb*1 gene in mice leads to transformation of the r4 territory into an r2-like state [29], whereas retroviral-mediated over expression of *Hoxb*1 in r2 causes homeotic transformation of r2 to an r4-like condition [30].

Evidence is now also emerging about some of the other factors that act upstream of Hox genes themselves to activate them at appropriate AP levels. As well as cross-regulation between the various Hox genes, upstream regulators of the Hox genes include Mafb and Krox20 which, in addition to controlling segmentation of the neuroepithelium act in a parallel but related process to regulate the Hox genes. Thus, Mafb directly modulates expression of paralogue group 3 Hox genes in r5 [31,32], and Krox20 is a direct activator of both *Hoxa*2 and *Hoxb*2 [33,34] and a repressor of *Hoxb*1 [35].

Given the prevalence of transcriptional regulation in the specification of AP pattern of the neuraxis, the aim of this study was to identify novel candidate or known genes with potential roles regulating the AP pattern of the neural tube. Many methodologies have been developed that are designed to identify and/or sample the array of genes expressed by a cell or tissue type, commonly defined as the transcriptome. The technologies vary both in the number of genes they are designed to profile and if *a priori *knowledge of gene identity is required. For example, technologies employing subtractive hybridization of cDNA libraries have been successfully used to find regulated changes in gene transcription during development of the neural tube [36]. A current method of choice is microarrays. However, due to the lack of availability of chick-based systems at the time of onset of this study, we chose to implement a large-scale messenger mRNA differential display screen [37]. Despite a prevalent false positive rate, recent advances in primer technology, gel electrophoresis and cDNA cloning of the appropriate differentially expressed gene has dramatically decreased such error [38]. An advantage of DD is that it can be used simply and relatively cheaply to assay the gene expression profiles of multiple cell or tissue types simultaneously, to detect both up and down transcriptional regulation. When taken together with the capacity to precisely stage and dissect the developing chick embryo the methodology presents a unique opportunity to accurately assay transcriptional regulation of the early CNS. In addition, despite the prevailing idea that all genes can be identified computationally once a genome has been sequenced, there is now clear evidence that a substantial amount of genome annotation is still required and novel genes are still being identified by other approaches [39,40]. Thus, there is still good reason to find novel genes using empirical screening technologies and place them into an appropriate biological context.

We present the findings of an exhaustive differential display screen for regionally restricted transcripts during early brain development. Clones were sequenced and compared to the latest EST, genomic, known gene and predicted gene databases to characterize differentially expressed candidates. Results were confirmed by in situ hybridization and the embryonic expression of several of the isolated genes is described for the first time. Taken together this study provides a foundation from which genetic networks and hierarchies can be inferred and further explored.

## Results

### Efficacy of the messenger RNA differential display screen

Preliminary data from this screen was reported in (42). We now report the results from the completed screen as well as presenting analysis, identity and expression for many of the candidate cDNAs isolated. We have used all 240 primer combinations available (12 (dT)_12 _T7 anchor primers in combination with 20 arbitrary primers; Hieroglyph kit, Beckman Coulter), as well as including some custom arbitrary primers (see Experimental procedures). According to manufacturer's data, this approach should survey >95% of the transcriptome. We recorded 44 reproducible changes in differential display gel profile from the 300 primer combinations tried in total (Fig. 2A). The changes observed were robust in that they appeared in the series 1 and 2 RNA pools and were reproducible in subsequent identical differential display experiments (data not shown). Overall, this represents a 14% incidence of difference in gene transcription across neural tube regions tested. The clone designation, identity, function and recorded gel profile of each of the differentially expressed clones are listed in Table 1. Fig. 2B shows a schematic representation of the molecular functions of the differentially expressed candidates whilst Fig. 2C shows how the changes were distributed across the neural tube. A large proportion of the changes were recorded as being present in the midbrain or rhombomere 1 regions (17 of the 44) which is consistent with this being previously known to be a site of active transcriptional events. Patterns of gene expression unique to certain areas were also seen which is compatible with prior knowledge of patterning of that region (e.g. hindbrain-specific). Other profiles were also present involving combinations of transcriptional activation (e.g. On in forebrain, midbrain and hindbrain but off in R1; others 10 of 44).

**Figure 1:**
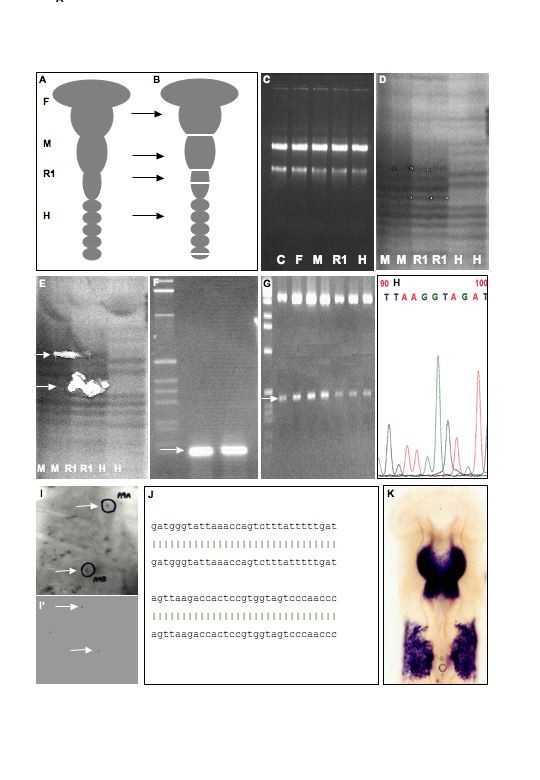
**Schematic diagram showing the sequential stages in the messenger RNA differential display screen**. **A**. HH st10 chicken embryos were harvested from the eggs and neural tubes dissected as shown in (**B**). **C**. Prior to use, total RNA integrity of forebrain, midbrain, rhombomere 1 and hindbrain samples were compared to RNA extracted from an un-manipulated HH st10 chick embryo (lanes 1 [control; C] vs 2–5). **D**. RNA extracted from F, M, R1 and H samples was subjected to differential display RT-PCR as described in Materials and Methods. **E**. Differentially expressed cDNA bands were cut from the gel and re-amplified. **F**. Agarose gel electrophoresis of reamplified band obtained in (E). **G**. Each re-amplified cDNA was cloned in the TOPO TA vector (Invitrogen, USA). A minimum of six independent clones from each sub-cloning were picked and sequenced **H**. The differential display fragment was used a probe to isolate a longer clone from a chicken HH st10 cDNA λZAP library by primary (**I**) and secondary (**I'**) screening. The resulting library cDNA was released from the λ vector using manufacturer's instructions, sequenced on both strands and identity was investigated by comparison to sequence databases (**J**). **K**. Expression of the clone was determined by *in situ *hybridization to HH st10 chick embryos (rostral is at the top of image). **F **= forebrain, **M **= midbrain, **R1 **= rhombomere 1, **H **= hindbrain (rhombomere 2–7).

**Figure 2:**
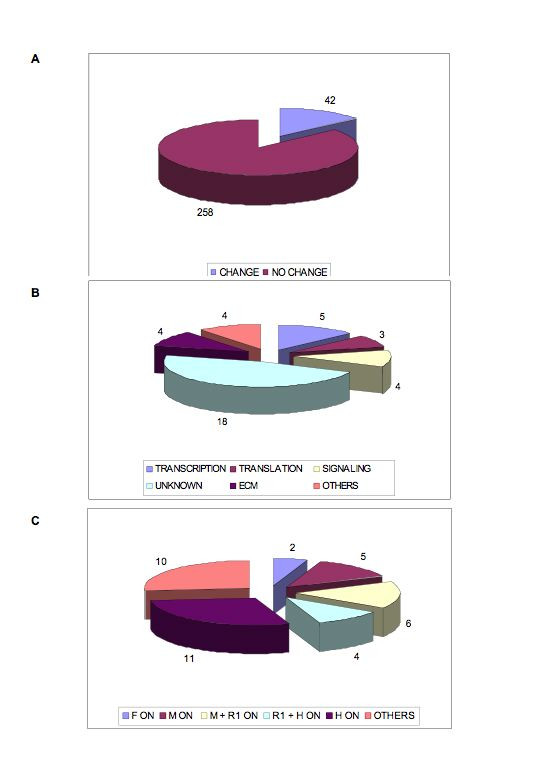
**Schematic representation of the data collected from the mRNA differential display screen**. **A**. From the **300 **primer combinations used **44 **differences in gene expression were recorded. **B**. Functional classification of the **44 **cDNAs. **C **A depiction of the pattern of transcriptional profiles identified in the screen. **F **= forebrain, **M **= midbrain, **R1 **= rhombomere 1, **H **= hindbrain (rhombomeres 2–7).

**Table 1:** Summary of cDNA fragments isolated by differential display of mRNA from fore/mid and hindbrain regions of a HH st10 chick embryo

**Clone**	**Accession**	**BBSRC**	**Encoded**	**Function**	**Remark**
A	Q05916 [SP]	603103425F1	Engrailed 1	TF	See figure 3A
B	No match	603743047F1	mafB	TF	See figure 3K
C [x3]	Bankit754392	603407923F1	SPARC-related 1	Cell adhesion	See figure 3H 2.8 kb cDNA from 5' RACE
D	gi|4929813	603118757F1	Sprouty2	Intracellular signaling	See figure 3B [42]
E	gi|17440991	603139218F1	KIAA0007	Unknown	See figure 3N
F	Bankit754394	No match	Similar to HB GAM*	Heparin-binding	See figure 3K 3.2 kb cDNA from library
G	Bankit754396	603856249F1	Unknown	Unknown	See figure 3E 1.6 kb cDNA from library
H	Bankit754406	No match	Unknown	Unknown	DD: Up in Midbrain
I	gi|12407844	603127196F1	Peroxiredoxin	Redox signaling	DD: Up in Midbrain
J	Bankit754410	No match	Unknown	Unknown	DD: Up in R1 & Hindbrain
L [X2]	gi|12729652	603488169F1	L1CAM	Cell adhesion	See figure 3F
N	Bankit754412	No match	Unknown	Unknown	DD: Up in Hindbrain
O	No match	602554245F1	Unknown	Unknown	DD: Up in Hindbrain
P	Bankit754414	No match	Unknown	Unknown	DD: Up in Hindbrain
Q	gi|1710781	604136738F1	LRP37/p40	Cell adhesion	See figure 3M
S	gi|550024	603129809F1	Ribosomal protein	Translation	DD: Up in R1 & Hindbrain
U	Bankit754420	No match	Unknown	Unknown	DD: Up in R1 & Hindbrain
V	gi|12653908	603762906F1	exoribonuclease	RNA processing	DD: Up in Forebrain
W	gi|13489085	603493341F1	Ubiquitin-conjucating enzyme	Protein turnover	DD: Down in Midbrain
X	Bankit741871	No match	Unknown	Unknown	DD: Up in Hindbrain
Y	Bankit754720	No match	Unknown	Unknown	DD: Up in Hindbrain
Z	Bankit741629	No match	Unknown	Unknown	DD: Up in R1 and Midbrain
β	Bankit754422	603124149F1	Pax2*	TF	See figure 3D 2.8 kb cDNA from library
1	gi|550024	603129809F1	ribosomal s10	Translation	DD: Up in Midbrain
2	gi|441123	603788872F1	RTPzeta	Intracellular signaling	See figure 3C
3	Bankit741889	No match	Unknown	Unknown	See figure 3I
4	Q90835 [SP]	603120688F1	EF1α	Translation	See figure 3J 2 kb cDNA from library
5	Q90749 [SP]	603848654F1	FGFR2	Intracellular signaling	See figure 3G [56]
6	No match	603408919F1	Unknown	Unknown	DD: Up [weak] in Midbrain
7	gi|14211561	603373072F1	GLP1	G-protein signaling	DD: Up in R1 & Midbrain
8	gi|15281514	603217934F1	Roundabout1	Axon guidance	DD: Up in Hindbrain
9	AAK55455	604170469F1	hypothetical protein 127.7 kDa	Putative TF regulator	DD: Up in R1 & Midbrain
10	No match	603107258F1	Unkown	Unkown	DD: Up [weak] in Hindbrain
11	Q9Y2L0 [SP]	603499417F1	KIAA1007 protein	Unknown	DD: Up [weak] in Midbrain
12	Q9NR13 [SP]	603951558F1	ALR-like	cytokine	DD: Up [weak] in Midbrain
13	Q9P278 [SP]	603610122F1	KIAA1450 protein	unknown	DD: Up in R1 and Hindbrain
14	No match	603005063F1	unknown	unknown	DD: Up in Fore and Midbrain
15	P08125	603848942F1	Collagen Alpha 1[X]	connective tissue	DD: Up in Hindbrain
16	X67778	604141228F1	Claustrin	Cell adhesion	DD: Up in F & M
17	No match	No match	EF2^+^	Translation	DD: H on
18	No match	603401645F1	EiF3zeta	Translation	DD: R1 & H on

Where an unequivocal identity is found for the differential display clone in BBSRC, Genbank or SWISSPROT databases, it is shown in the appropriate column. SP = Swiss-Prot Database, TF = Transcription Factor, RTP = Receptor Tyrosine Phosphatase, FGFR = Fibroblast Growth Factor Receptor, GLP = Glugacon-like Peptide, EF = Elongation Factor, LRP = Laminin Receptor Protein, L1CAM = L1-like Cell Adhesion Molecule, mafB ~ kreisler, 5' RACE = rapid amplification of 5' cDNA end. Where no identity for a cDNA is found in the sequence repositories, the sequence is given in Supplementary Information. **F **= forebrain, **M **= midbrain, **R1 **= rhombomere 1, **H **= hindbrain (rhombomeres 2–7). * denotes clones whose identity has been derived from significant matches with the chick genome sequence (see Results). ^+ ^denotes identity derived from similarity to an ortholog. Where a 100% match with an EST from the UMIST collection was found the fragment sequence was not submitted to Genbank.

### Coverage of messenger RNA screen

**Figure 3:**
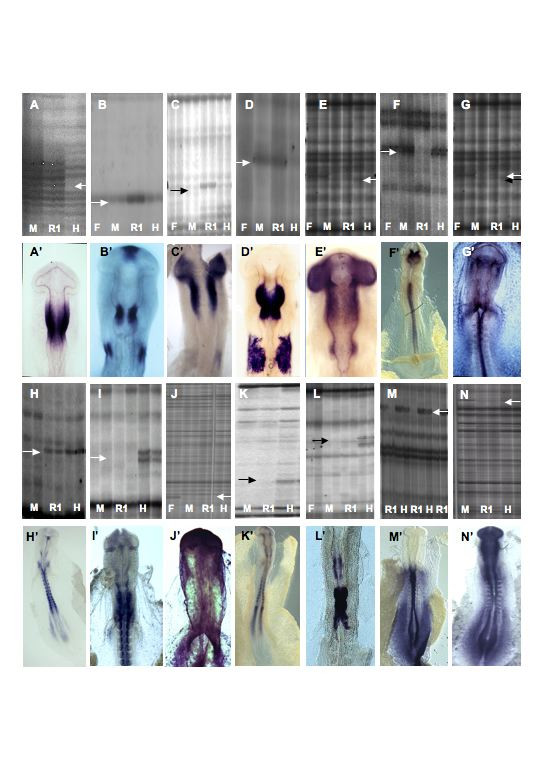
**Differential display gel profile and corresponding chick embryo whole mount *in situ *hybridization**. (**A, A'**) Clone A (250 bp; Engrailed 1). (**B, B'**) Clone D (700 bp; Sprouty 2). (**C, C'**) Clone 2 (560 bp; RTPzeta). (**D, D'**) Clone β (440 bp; Pax 2: genome-derived identity). (**E, E'**) Clone G (406 bp; Unknown). (**F, F'**) Clone L (220 bp; L1CAM). (**G, G'**) Clone 5 (450 bp; FGFR2) (**H, H'**) Clone C (525 bp; similar to SPARC related protein 1: genome-derived identity). (**I, I'**) Clone 3 (660 bp; Unknown). (**J, J'**) Clone 4 (257 bp; EF1α). (**K, K'**) Clone F (586 bp; similar to HB-GAM: genome-derived identity). (**L, L'**) Clone B (600 bp; MafB). (**M, M'**) Clone Q (1700 bp; LRP37/p40) (**N, N'**) Clone E (1200 bp; KIAA0007). In all cases the observed expression pattern correlates with that expected from the differential display gel profile. **F **= forebrain, **M **= midbrain, **R1 **= rhombomere 1, **H **= hindbrain (rhombomeres 2–7). The arrow (black or white) denotes the position of the differentially expressed PCR product. The size of the original PCR product is given in base pairs (bp). See also Table 1.

A differentiated eukaryotic cell is thought to contain 12,000–19,000 distinct mRNA species (Liang and Pardee, 1992; DC and A Lumsden unpublished observation). In the study described here 300 primer combinations were used, each displaying around 100 PCR products (data not shown) to generate over 30,000 amplified fragments. This figure broadly correlates with the current estimate of gene number for a higher eukaryotic organisms [39,40]. Thus, in principle we surveyed the majority of genes expressed during the transcriptional specification of the fore-, mid- and hindbrain regions of the developing avian CNS. However, the total number of bands displayed does not compensate for the observed redundancy of the displays. That is, many mRNAs would have been displayed more than once, whereas others, particularly low abundance messages, would not have been detected at all. This is an intrinsic property of the differential display methodology and contributes to the restricted coverage generated. An example of redundancy was observed with Clone C, where the same differential display gel profile (i.e. switched on in r1 and hindbrain samples but off in forebrain and midbrain; Fig. 3H') and subsequent cDNA identity was observed from candidate bands of a different size obtained using a different arbitrary primer (data not shown; Table 1). By contrast, a slight under-estimation of the number of distinct PCR products is possible due to the existence of more than one species of PCR product of the same size in an individual band (data not shown). In these circumstances, a Single Stranded Conformational Polymorphism (SSCP) strategy was adopted to accurately determine the differentially expressed cDNA (data not shown). An additional consideration to the restricted coverage of the technique is that where a fixed number of PCR cycles are used (such as in this entire study), it is likely that small differences in the levels of some regulated transcripts will be missed [44].

### Compiling identities of the chick differential display cDNA clones

Of the 44 differentially expressed genes identified, 27 could be assigned an unequivocal identity either directly from the sequence of the displayed fragment or following the isolation of a longer cDNA clone. For the remaining clones, often after the addition of more upstream sequence from cDNA libraries, an identity, putative function or membership to a gene or protein family could not be assigned. It is likely that, despite our efforts to isolate large cDNA fragments, the 3' bias of the methodology resulted in preferential isolation of 3' untranslated (3'UTR) sequences. For many clones, the recent release of the chick EST database was similarly insufficient to assimilate an identity. The chick ESTs were generated from 21 different embryonic and adult tissues, ranging from a complete early developmental stage HH st10 embryos to a single adult tissue e.g. the limb. Despite the fact that the individual cDNA libraries constructed from each point were poly T-primed and therefore generated from the 3' end of an mRNA transcript, a similarity of the 312,000 usable EST sequences with the clones described here was not always found. This may be a function of the representation of the EST libraries or that only the 5' ends of the directionally cloned libraries have so far been sequenced (see [52]) Thus, for those longer clones in the EST repository, the sequence immediately upstream of the poly A tail may be under represented. These types of issues also imply that studies like those described here have a useful role in annotating genomes and contributing to an accurate description of transcriptomes.

In the absence of a similarity match from the cDNA databases, we searched the recently released chick draft sequence (Build 1.1; University of Washington) for potential information (82). Subsequent to a significant match (>98% identity) against the chick genome, we identified the appropriate sense strand and investigated up to 50 kb upstream for exons that might be informative. This approach, although helpful, has the caveat that associated exons must first be shown to physically spliced to the cDNA query sequence, for example by RT-PCR, before an identity can be unequivocal. Current data suggests that the average 3'UTR is within the range of 0.5 to 2.0 kb (83). Where possible, a Northern blot was performed to indicate the appropriate size of cDNA to look for in the genome. However, for some clones no matches could be obtained from the genome sequence confirming that sequence remains to be finalised.

### Validation of the experimental technique: isolation of En1, MafB, Spry2, FGFR2, Robo1

The strategy described here used a microsurgical approach and a subsequent pooling strategy prior to the screen. Proof of principle was confirmed by the isolation and identification of five genes known to be expressed in embryonic patterns faithful to the differential display gel profile and also to play important roles during neural tube development. Analysis of the display profile from the subset of mRNAs amplified with anchor and arbitrary primer 3 revealed a PCR product, clone A, that was reproducibly seen in the M and R1 samples but not in the F and H samples (Fig. 3A (F not shown)). Subsequent isolation, reamplification and cloning of this band gave a 250 bp cDNA sequence in all 6 clones tested. Comparison of this sequence using BLASTN showed it to be 100% identical to the 3' UTR region of chick Engrailed 1 cDNA (data not shown). Further confirmation was obtained by *in situ *hybridisation to chick HH st10 embryos where the expression pattern observed was identical that previously described. *En*1 has previously been reported as being a primary genetic determinant of the developing midbrain and rhombomere1 regions and mice homozygous for an En1 loss of function allele lack these entire structures [[53], reviewed by [8,21]]. Clone B derived from an anchor primer 8 – arbitrary primer 17 combination-generated 600 bp hindbrain-enriched PCR band (Fig. 3L), was found to be 94% identical to the 3'UTR of the mouse Mafb (*Kreisler*) cDNA. *In situ *hybridisation with this cDNA demonstrated that expression was fully concordant with the previously described expression in rhombomeres 5 and 6 in the HH st10 neural tube (Fig. 3L'). Available data demonstrates the developmental significance of this gene since animals lacking functional mafB protein show, amongst other developmental defects, a deletion of the rhombomere 5 region [54,55]. Additional evidence for the efficacy of the screen described here was obtained from the reamplification, cloning, sequencing and expression analysis of clone 5 that displayed a midbrain-enriched gel profile (Fig. 3G,G'). This 450 bp clone showed 100% identity to the 3'UTR of the FGFR2, a transmembrane receptor tyrosine kinase that was previously described as being expressed in the midbrain vesicle of a HH st10 chick embryo [56]. The cloning, expression and interplay with FGF8 signalling of sprouty2 (an intracellular antagonist of FGF-signalling; clone D) was also reported from this screen [[42]; reviewed by [57]]. A detailed functional analysis of the role of sprouty proteins in development is being performed in our laboratory (e.g. see [58]). Taken together, these data demonstrate that the screen was reflective of the transcriptomes surveyed and was capable identifying a range of genes with varied and important development roles. As such, the data presented in Table 1 is likely to describe a true picture of gene expression changes in the developing neural tube.

### Differential display has an efficacy lower than that statistically predicted

Despite the established efficacy of the screen, the detection of fewer than 50 differentially expressed bands is indicative of a relatively small proportion of mRNAs with altered expression across the AP neuraxis of a HH st10 developing chick embryo. That we did not identify any of the large number of previously characterised differentially expressed genes associated with hindbrain patterning (e.g. the *Hox *genes), strongly suggests that the coverage was less than anticipated. This may be due in part to the low level of mRNA abundance of the Hox transcription factors. However, this is largely countered by the observation that they can be readily detected by wholemount *in situ *hybridisation. A survey of the accumulated literature shows that greater than 150 genes have been reported as being differentially expressed in the tissues under study here [e.g. see [26,36,59,23]]. It is clear from this study that messenger RNA differential display operates at efficiency much lower than that statistically predicted by binding of the arbitrary primers to their cognate sequence alone.

### Detailed expression analysis

Following validation of the screen, we selected several identified genes for detailed expression analyses; SPARC-related modular calcium binding protein 1, Laminin Receptor Protein 37, Clone β, HB GAM, Clone G, Translation elongation factor 1α, Receptor protein tyrosine phosphatase zeta and L1CAM. These clones were selected based upon their identity and possibility that they may interact with other known pathways that pattern the mid-hindbrain territories at this developmental time; For example, to ask if RTPζ and HB GAM are expressed in the appropriate place to modulate/interact with FGF signalling around the isthmus organiser? Although others showed no obvious identity (e.g. clone G and β), they were selected based upon their differential display profile (e.g. enriched in M & R1) that suggested that they were expressed in domains known to be important in patterning the areas under study (e.g. the mid-hindbrain boundary). Similarly, we chose clones who have a know role in cell adhesion to see if they could contribute to either boundary formation or cell lineage restriction in the mid-hindbrain region. In addition, for some of the clones isolated it was not possible to produce a definitive in situ hybridisation pattern. This may be due to the 3' UTR nature of the sequence. The identities of the cDNAs not used for further in situ hybridisation studies, where known, is presented in Table 1.

### Clone C: SPARC-related modular calcium binding protein (SRMCBP) 1

**Figure 4:**
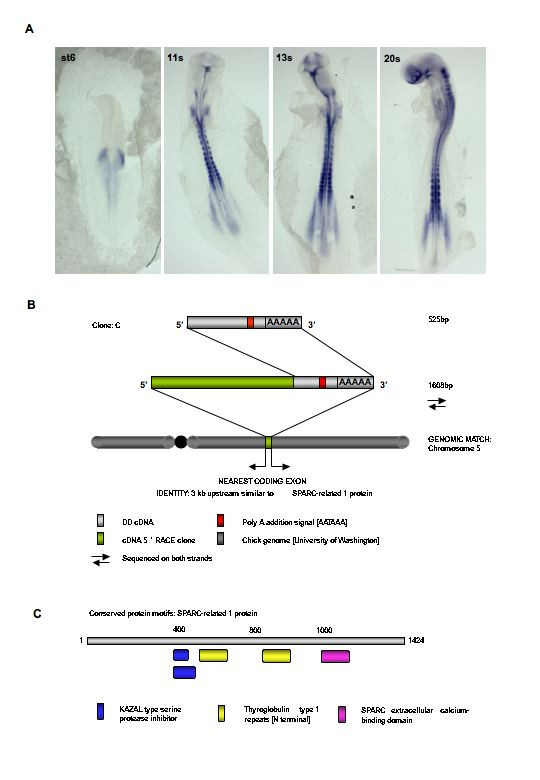
**Identification and expression of SPARC-related 1**. **A**. Time course expression of SPARC-related modular calcium binding protein 1 in the developing chick embryo. **B**. Summary of the differential display cDNA and library clones isolated for clone C. Representation of the match (98%) obtained from the chick genome and subsequent identity derived; similar to SPARC related protein 1. DD = differential display cDNA

Using arbitrary and anchor primers 7 and 3 respectively, as well as 5 and 3 respectively, a 525 bp PCR band was reproducibly enriched in the series 1 and 2 R1 and H mRNA pools. Reamplification, cloning and sequencing of the cDNA clone on both strands failed to produce a significant match within the databases. The 3'UTR sequences showed a typical poly A addition consensus site (AATAAA) proximal to the site of anchor primer binding (the poly A tail). This clone was not represented in either of the cDNA libraries screened. Using a nested 5' RACE approach, a further 2.3 kb of sequence immediately upstream of this 3'UTR sequence was acquired. This sequence lacked any obvious ORFs, partial or otherwise (data not shown), and no identity was derived suggesting that it was the 3'UTR region. Scanning of the chick genome with clone C sequence revealed a region of 98% (2714/2838) identity on chromosome 5 (NW_060388.1). Immediately adjacent to this match, exist a previously annotated protein coding exon coding for 'similar to SPARC-related modular calcium binding protein 1' (Genbank XP_426431; GI 50748984) (Fig. 4B). The immediacy of the clone C-derived sequence to this region implies that clone C codes for the protein described as for 'similar to SPARC-related modular calcium binding protein 1'. SPARC (BM-40) has an extracellular Ca^2+ ^binding domain (containing 2 EF-hand motifs) and is a multifunctional glycoprotein that functions to regulate cell-matrix interactions. It binds to such proteins as collagen and vitronectin and can binds to endothelial cells and inhibit cellular proliferation. The extracellular (EC) domain interacts with a follistatin-like (FS) domain that appears to stabilize Ca2+ binding. The two EF-hands interact canonically but their conserved disulfide bonds confer a tight association between the EF-hand pair and an acid/amphiphilic N-terminal helix. Proposed active form involves a Ca2+ dependent symmetric homodimerization of EC-FS modules (see Fig. 6C for conserved domains) (reviewed in [60]).

SRMCBP1 has dynamic expression in the developing embryo (Fig. 4A); expression is first seen in the neural plate followed by staining in the primitive streak. At the onset of somitogenesis, SRMCBP1 becomes restricted to presumptive tail bud and newly formed somites. Later at 3–6s, the transcripts are more abundant in lateral tail bud regions but still present in the somites. At 12s, SRMCBP1 transcripts are seen in rhombomere 2, ectodermal regions surrounding the otic vesicle and medial somites. At HH st16 the hindbrain expression has expanded into all rhombomeres but is excluded from the boundary zones.

### Clone Q: Laminin Receptor Protein (LRP) 37

**Figure 5:**
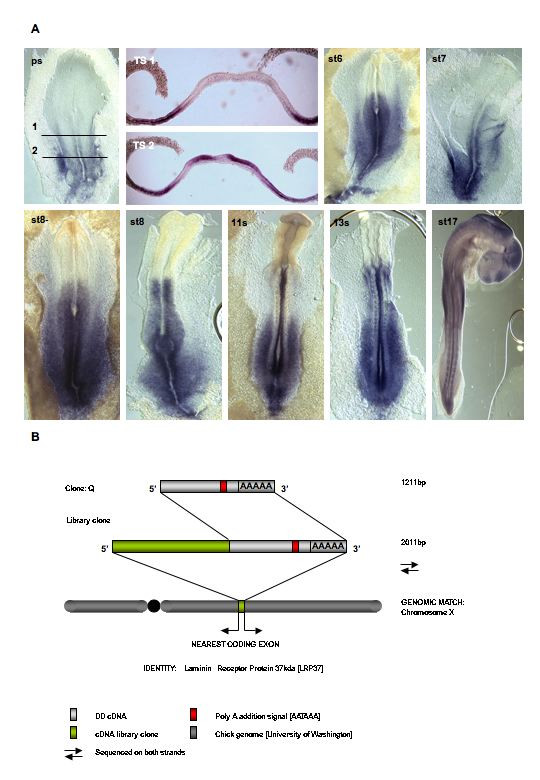
**Identification and expression of LRP37/p40**. **A**. Expression of clone Q (LRP37/p40) in the developing chick embryo **TS **= transverse section **B**. Summary of the different display cDNA, library and genomic information used to attain the identity of Q. **B**. Summary of the differential display cDNA and library clones isolated for clone C. Representation of the match (100%) obtained from the chick genome and subsequent identity derived; similar to LRP37/p40. DD = differential display DNA

Analysis of the differential display gel profile obtained using anchor primer 7 combined with arbitrary primer 7 consistently and reproducibly identified a cDNA of 1700 base pairs in series 1 and 2 mRNA pools (Fig. 3M&M') whose expression was switched on in the posterior hindbrain cDNA pool but not in the rhombomere1 cDNA pool. Excision of this candidate cDNA, cloning, library screening, sequencing and BLAST analysis revealed it to be 100% identical at the nucleotide level to the previously identified *Gallus gallus *Laminin Receptor Protein 37 gene (Fig. 5B). The laminins are a family of glycoproteins that form a critical component of the basement membranes of most organisms and are known to be an important factor in cell migration, axon pathfinding and modulation of cell survival as well as other cellular processes. Laminins self assemble from α, β and γ subunit chains and are secreted into the extracellular space where they can interact with a range of other extracelluar matrix molecules. To date, twelve different laminin heterotrimers have been identified in mammals as well as other variants being found in Hydra, Drosophila and C. elegans. It has been reported that the laminin family members have tissue specific, but overlapping, distributions during embryonic development (see [61] for summary). Critically, laminins have been shown to have a diverse and important and role during embryonic development to later post natal development. For examples and a recent review see (61). The primary mechanism by which cells recognize and interact with laminins is through the integrins, a family of heterodimeric transmembrane receptors consisting of α and β subunits. Integrins are capable of exerting regulatory effects on both the cytoskeleton and cellular signaling apparatus.

LRP has been described as a multifunctional molecule involved in both cell adhesion via laminin and formation of the polysome in the cytosol. *In situ *hybridisation reveals that the LRP transcripts are expressed very early in embryonic development in regions fated to form mainly trunk structures. As development proceeds, the expression of LRP is observed in the neural tube, somites, ectodermal and endodermal tissues, but only immediately posterior to the rhombomere (r) 6/7 border. Interestingly, this gene has not been previously reported to be expressed embryonically and its function in development remains to be elucidated (Fig. 5A)

### Clone β: Pax2

**Figure 6:**
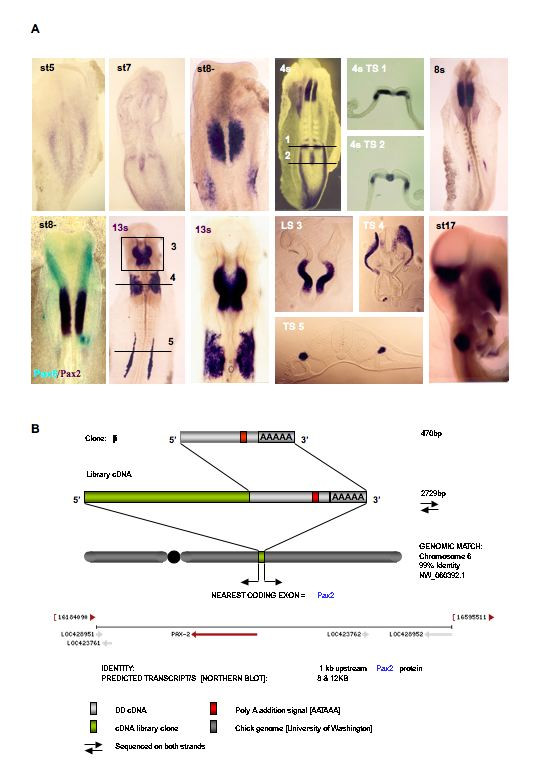
**Identification and expression of Pax2**. **A**. Expression of clone β/Pax2 in the developing chick embryo. **TS **= transverse section **B**. Summary of the different display cDNA, library and genomic information

Given the differential display gel profile of clone A and its identification to be the developmentally significant En1 cDNA, it was of particular interest that clone β had an identical gel profile (Fig. 3d). Reamplification, cloning and sequencing produced 8 identical subclones that gave 440 bp of sequence. Similar to other clones described here, this clone gave no identity or no expression pattern in HH st10 chick embryos. A high stringency library screen of the HH st10 chick λZAPII library produced 3 independent clones ranging in size from 2 to 2.8 kb (data not shown). The longest of these clones was selected and sequenced on both strands. Despite the large amount of information no clear identity or ORF greater than 70 amino acids could b obtained. To determine the size of the endogenous transcript we performed a northern blot on chick whole embryo HH st10 total RNA and observed two bands of ~8 and 12 kb (data not shown). Given the size of the full length mRNA, it was likely that our sequence information may still have been in the 3'UTR. Using the elongated 2.8 kb clone β, we obtained the expression during the formation of the neural tube (Fig. 6A). Notably, the expression observed in the neural tube was similar that seen for clone A (*En1*) and consistent with the gel profile. In fact, the expression of clone β was recorded very early in the ectoderm destined to become the MHB.

To ascertain the identity of clone β and discern the potential early function in MHB formation, we scanned the genomic sequence upstream of the existing 2.8 kb. Using this approach we located an exon that codes for a region of the paired homeodomain transcription factor *Pax2*. This observation is consistent with the known *Pax2 *that is the earliest transcription factor expressed in the hierarchy of MHB formation (see [62] and references therein for example; reviewed by [21]). However, although clone β is now known to represent the previously well-characterized *Pax2*, it should be noted that the 2.8 kb *in situ *fragment provides extremely definitive patterns that may be useful in future studies. The methodology described here also serves as model to elucidate the identity of other cDNAs.

### Clone F: similar to heparin-binding growth-associated molecule (HB GAM)

**Figure 7:**
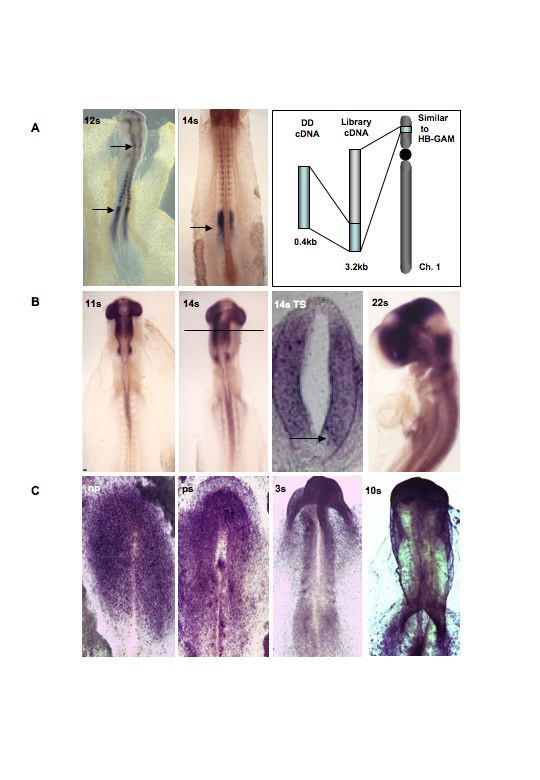
**Identification and expression of HB-GAM, clone G & Ef1α**. **A**. Expression of clone F (similar to HB-GAM) in 12 and 14s chick embryos including the strategy employed to attain its identity. **B**. Expression of clone G 12s, 14s, and 22s chick embryos including a transverse section at the midbrain level of 14s embryo. Expression is uniform throughout the DV axis but missing from the floor- and roofplate. **C**. Expression Ef1α from neural plate to 10s stage embryo. Transcripts are enriched in the rostral part of the embryo.

Clone F is a reproducibly expressed hindbrain-enriched 586 bp display fragment. The available sequence was insufficient to discern identity or expression for *in situ *studies. Following screening of the chick HH st10 λZAPII cDNA library, four independent clones were obtained. Verification of clone F identity by southern blotting (data not shown) and sequencing revealed an additional 2.5 kb of sequence had been attained. The largest construct showed no extended open reading frames or BLASTN/X identity with the existing sequence warehouses. However, submission of the extended clone F sequence to the chick genome BLASTN algorithm revealed a 97% identical match to the NW_060209.1 contig derived from chromosome W sequencing (Fig. 7A). Further analysis of this locus (LOC418125) showed the presence of the previously annotated gene defined as 'similar to heparin-binding growth-associated molecule (HB GAM)' (also known as similar to Pleiotrophin precursor (PTN) (Heparin-binding growth-associated molecule) (HB-GAM) (Heparin-binding growth factor 8) (HBGF-8) (Osteoblast specific factor 1) (OSF-1) (Heparin-binding neutrophic factor) (HBNF)), immediately adjacent to the clone F sequence match. Given the appropriate orientation and 3'UTR nature of the clone F sequence, it is reasonable to conclude that clone F is the gene described as 'similar to HBGAM'.

Heparin-binding growth-associated molecule itself is a basement membrane-associated protein that was initially isolated as a neurite outgrowth-promoting factor from perinatal rat brain [63,64]. HB-GAM is composed of two thrombospondin type 1 (TSR) domains flanked by lysine-rich N- and C-termini of undefined structure. The TSR domains may mediate binding to heparan sulfates and to the cell surface, while the role of the lysine tails is unknown [65]. HB-GAM also has another close relative; midkine (MK) that was isolated and cloned as a retinoic acid induced differentiation factor [66]. However, although the *in vivo *functions of HB-GAM are still not generally understood, recent work by [67] has demonstrated a clear role for HB-GAM in the selective inhibition of FGF2-mediated activation of FGFR1 by restricting the interaction between ligand and the proteoglycan co-receptors required for normal activation (see [68] for review of FGF signalling). In this context, HB-GAM inhibited the proliferation and promoted the differentiation of neural stem cells by restricting the signalling activities of FGFs.

The capacity of HB-GAM (or closely-related proteins) to negatively regulate FGF-stimulation of their cognate receptors is of significance given the expression of clone F in the developing embryo. At HH st11- clone F transcripts are seen in rhombomere 3, medial somites and the presomitic mesoderm (Fig. 7A). The pattern observed in the presomitic mesoderm is of particular interest as it is known that FGF8-mediated signalling is required here to maintain proliferation and inhibit inappropriate maturation of this zone (reviewed [69]). Thus, the FGF8-mediated presomitic signalling pathway may be mechanistically regulated by HB-GAM (or the 'similar to HB-GAM' equivalent) in an analogous fashion to that described by (67). We are currently conducting a series of functional interference studies to determine the precise role of this protein during early patterning of the neural tube.

### Clone G: unknown

Amplification of the mRNA pools with anchor primer 8 and arbitrary primer 15 revealed a 406 bp PCR product whose expression was recorded principally in the forebrain, midbrain and rhombomere 1 samples but absent from the hindbrain group. Sequence analysis followed by comparison to databases showed no significant homologies. Using this cDNA fragment as a probe, 3 independent positive plaques were isolated from the chick HH st10 λZAPII cDNA library. Excision of the insert and subsequent southern blot analysis determined the longest of these clones to be ~1600 bp. The sequence of this clone is presented in Supplementary information and was shown to be 100% identical to the clone G probe sequence at the 3' end. The extended clone G sequence provided no further insight into the identity of the resultant protein. The UMIST chick EST database does not contain the equivalent cDNA sequence. Furthermore, this sequence is as yet unrepresented in the available draft of the chick genome. However, northern analysis shows that for total RNA derived from a HH st10 embryo that there are two transcripts of equal abundance at 4.1 and 4.5 kb (data not shown). Thus with 1.6 kb of sequence and the expected size of the coding RNA, the open reading frame and potential function can be predicted upon completion of the genome sequence.

Using the longest library clone to generate an antisense probe, the expression of clone G in a chick embryo was shown to mirror the differential display gel profile (Fig. 7B). At HH st 10+, clone G transcripts were seen in the forebrain, midbrain and rhombomere 1 regions but completely absent from the hindbrain (r2-8) region of the neural tube. Notably, there was an obvious gap in the expression at both the anterior neuropore and MHB. These are both sites of FGF8-signalling and raise the possibility that clone G is repressed by FGF8. This supported by the observation that there are no gaps between the zones of expression of Fgf8 and clone G as assessed by a single colour 'fill in' *in situ *hybridization (data not shown). The capacity of FGF8 protein to repress the transcription of clone G is currently under investigation using FGF8-coated beads implanted into the anterior midbrain. Transverse section of HH st10+ embryos in the rostral midbrain show that expression in the DV axis is excluded from the roofplate and floorplate areas (Fig. 7B) suggesting that clone G may also be repressed by factors expressed in these regions such as BMPs and SHH respectively.

### Clone 4: translation elongation factor 1α

Using a similar cloning and sequencing approach to that described elsewhere, we demonstrated that a 257 bp PCR product enriched in the forebrain pools corresponded to a 2011 bp library cDNA with 100% identity to *Gallus gallus *translation elongation factor 1α (EF1α}. Given that EF1α is often used as a control for a ubiquitously expressed gene, it was somewhat surprising to find a gel profile suggestive of restrictive distribution. Using the longer cDNA, the expression of EF1α in embryos was observed to mimic that predicted by the gel-banding pattern (Fig. 7C). Even at early stages, EF1α are more abundant in the anterior neural plate. As development proceeds up to HH st10, EF1α expression becomes most evident towards the rostral end of the embryo. The function of this type of pattern remains to be determined.

However, there is now increasing evidence that mRNA-specific translational control, whereby the translation of a defined group of mRNAs is modulated without affecting general protein biosynthesis or the translational status of the cellular transcriptome as a whole is an important factor in the regulation of cellular identity. In addition, global control of protein synthesis can also be achieved by changes in the phosphorylation state of initiation factors or the regulators that interact with them (reviewed by [70]). In addition, previous studies have also implicated the differential distribution of protein coding machinery in the developing neural tube. The translational-regulatory component eIF2a kinase-related is expressed exclusively in r5 of the developing hindbrain [36]. Here, a potential role in the r5-specific regulation of apoptosis has been speculated. It is of note that the two translation factors reported here, EF2 and Eif3zeta, showed a robust hindbrain-specific gel profile (Table 1). Taken together, these data are suggestive of a neural tube specific combinatorial translation factor code operating alongside the well-established transcription factor patterning events.

### Clone 2: receptor protein tyrosine phosphatase zeta

Previous work reported from this study [42,71] has helped to elucidate some of the molecular regulation of FGF-signaling at the MHB. It was therefore of interest when the identity and expression of clone 2 was elucidated. Using the ACAATTTCACACAGGATTTTGGCTCC arbitrary primer in conjunction with GGAAAAAAAAAAAGCCCTATAGTGAGTCGT anchor primer, a 560 bp PCR consistently upregulated in midbrain and forebrain pools of series 1 and 2 mRNAs was obtained. Reamplification, cloning and sequencing of the differential display band followed by database queries produced no clear identity. To establish an identity we submitted the sequence to the chick EST database and commenced a 5' walking strategy [52]. This approach gave an exact match to the 603788872F1-designated assembled sequence contig . Using the longer sequence an unequivocal match (100%) to *Gallus domesticus *phosphotyrosyl phosphatase mRNA complete cds (gi|441122|gb|L27625.1|CHKPHOPHOS) was found, which itself is othologous to the *Rattus norvegicus *receptor type protein tyrosine phosphatase zeta/beta (gi|1581693|prf||2117212A). Thus it has been established that clone 2 is chick ortholog of the receptor type protein tyrosine phosphatase zeta/beta.

Receptor protein tyrosine phosphatases (RPTPs) constitute a large family of structurally related proteins that are responsible for the regulated removal of phosphates from tryrosine residues. Tyrosine phosphatases are classified into 8 subfamilies spread across three groups: cytoplasmic, receptor-type, and dual specificity phosphatases that dephosphorylate serine threonine residues, and tyrosine residues that are in close proximity [reviewed by [72,73]] Receptor protein tyrosine phosphatase zeta is a member of the receptor type group with an extracellular domain similar to that of the cell adhesion molecules (CAMs) (Fig. 3C'). Evidence from genetic studies in *C. elegans *has demonstrated that one function of RPTPs is to antagonize receptor tyrosine kinase signalling. The RPTP, *Clr-1*, functions to inhibit signalling by the FGF receptor-related RTK, Egl-15, to regulate the migration of sex myoblasts [74]. In addition, a mitogen-activated protein kinase phosphatase (MAPK3) gene, encoding a dual specificity phosphatase, has been shown to directly antagonise FGF8-mediated receptor activation during limb development [75]. Given the potential function of clone 2 and a midbrain-enriched gel profile we selected this clone for further expression analysis by *in situ *hybridisation. Other than a report on the expression of RPTPγ [76], there is no description of the expression of RTPs in early embryogenesis

At HH st10+ (11s; Fig. 3C') the expression of RTPζ was observed in the midbrain and forebrain vesicles. The mRNA distribution in the midbrain is potentially in a caudal to rostral gradient. No expression was observed in the neural tube caudal to the MHB. Additional weak expression was also seen in the ectoderm immediately adjacent to r5 (otic ectoderm). Both the midbrain territory and otic ectoderm are known sites of FGF-signaling [reviewed by [21], see [77] (otic FGF-signaling)]. Thus we hypothesize that RTPζ may be involved in modulating the FGF-signaling events that are occurring in those regions. Alternatively, other than being involved in the control of FGF8-mediated signal transduction at the MHB, RTPζ may be involved in another independent process such axonal guidance in retinotectal mapping. The functional role of RTPs in these types of events has previously been documented [reviewed by [73] and references therein].

### Clone L: L1CAM

Data presented in Table 1 shows that following the library screening, sequencing and *in situ *analysis, clone L was the *Gallus gallus *close homolog of L1 cell adhesion molecule (L1CAM) with a distinctive transcript distribution. At HH10+, L1CAM was seen in the spinal cord, a caudal to rostral graded midbrain expression and some mRNA localized to the posterior forebrain. The gene expression pattern is fully correlative with the observed gel profile of on in all regions other the r1 pool (Fig. 3F,F'). L1CAM is a 200 kDa transmembrane cell adhesion molecule with six extracellular Ig-like and five fibronectin III domains that enable L1 to homophilically bind to opposing cells as well as to heterophilically bind to TAG-1, β1-integrins, F11/contactin, neural cell adhesion molecule (NCAM), and proteoglycans [reviewed by [78]]. L1CAM is capable of activating a MAP kinase signalling cascade through the intermediates Src, phospohinositide-3 kinase, Rac1, and p21-activated kinase, leading to neurite growth [for example see [79]]. Studies also suggest that retinal axons require the function of L1 in addition to repellent EphA guidance receptors to achieve proper topographic mapping [80] Thus this early, previously unreported, graded expression of L1CAM in the midbrain may play a role in establishing the appropriate projections of the retino-tectal system.

### A lack cDNA representation in current chick databases

The observation that neither the identity nor a clear wholemount expression pattern could be obtained from the differential display cDNAs cloned into the pCR-TOPO vector lead us to the search for longer more informative clones. However, for clones H, J, N, P, U, X, Y, Z & 3 no alternatives cDNAs were obtained from either screening of the chick λZAPII library, RZPD neural tube-specific gridded library , our in house 5' RACE library or the chick EST database (/chick) (Table 1). Together, this implies that these transcripts are expressed at a very low abundance or are refractory to traditional cDNA cloning methodologies. Furthermore, these sequences were not represented in the present draft of the chick genome sequence. Thus, following completion of the chick genome the identity and potential role of these genes in development can be re-assessed

## Discussion

Taken together, the confirmed expression of the known and novel genes describe here fully validates the efficacy and content of the screen (Table 1). Hence we provide a description of gene expression during early development of the neural tube that provides novel insights into some of the molecular pathways employed (e.g. extra- and intracellular signaling). The data provided here can thus be used as a foundation for future functional studies and valedictory support for forthcoming microarray studies. In particular, several of the potential patterning mechanisms alluded to here are being functionally pursued. For example, the midbrain-enriched expression of RTPz is being investigated for its capacity to negatively regulate isthmic-FGF8 signalling and its subsequent impact on midbrain patterning.

Using the latest advances in messenger RNA differential display technology described herein, as many as 500 different cDNAs were amplified per primer pair. An innate consequence of the technique is that any cDNA isolated, novel or otherwise, is anchored at the 3' poly A tail end of an RNA transcript. Given that the 3' untranslated (UTR) region of transcripts are generally greater than 500 bp and maybe up to 5 kb long (83), it has been traditionally difficult to obtain definitive information of a cDNA's identity. This has usually required a laborious cDNA-walking strategy to obtain further sequence information (e.g. **R**apid **A**mplification of **c**DNA **E**nds (5' RACE)). However, recent advances in whole genome sequencing and large scale EST projects have provided a large substrate of 3' sequence information that can be accessed and searched using standard BLAST methodologies. The availability of this resource has dramatically increased the ease with which an identity or probable function can be assigned to a cDNA obtained by DD.

Conversely, the work described in this study highlights a short-coming of differential display. Namely, this technique is restricted in the percentage coverage of the mRNA pool it samples. Using over 240 primer combinations (theoretically sufficient to investigate >90% of transcriptome) only 44 differentially expressed cDNAs were identified and characterized. This is at odds with previously known number of over 150 genes that are differentially expressed throughout the developing neural tube (for example see [36,26]). Therefore, DD in its latest guise has been shown to be capable of accurately and reproducibly monitoring gene expression, albeit in a restricted fashion. Using short cDNA sequences obtained from this screen, it has been possible to walk up to a further 3.5 kb 5' by reiterative BLAST comparison [52] with sequences deposited from the chick EST project (personal observation). Overall, the stringent technological advances and increased sequence data from genome wide studies have given DD a further lifespan as a useful cDNA screening technology for obtaining small numbers (typically <100; see Table 1) of differentially expressed genes in species where high density microarrays are not yet available

The data provided in this study will also be beneficial for the implementation and validation of the similar types of screens using the newly released chick high density oligonucleotide arrays (/products). Not only does it serve as a methodological platform for large scale screening purposes but also the gene expression data described provides an instant read out of the efficacy of the genechip approaches.

Despite the relatively restricted coverage of messenger RNA differential display, it still remains an extremely useful tool for the interrogation of transcriptomes. In particular for those species where high density microarrays are unavailable at present, it can be applied to the study of differential gene expression and used to isolate important regulatory transcriptional events. For example, in the quail retinoic acid-deficient system, (81) successfully used differential display to identify a large set of candidate genes whose expression was altered in the DV axis of the spinal cord compared to that of a normal spinal cord.

## Conclusion

This study provides an insight into some of the varied and novel molecular networks that operate during the regionalization of embryonic neural tissue and expands our knowledge of molecular repertoire used during development.

## Methods

### Embryo staging

Chicken embryos were collected from fertilised brown chicken eggs (Needles Egg Farm, Hertfordshire, UK) and staged according to Hamburger and Hamilton [41].

### Embryo collection and RNA extraction

To obtain sufficient total RNA for complete coverage of the available primer combinations (see Hieroglyph Kit, Beckman Coulter, USA) it was necessary to adopt a pooling strategy for the tissues. Individual embryo regions (Fig. 1A,B) were dissected using the characteristic morphological boundaries delineating the forebrain, midbrain and hindbrain compartments [42]. Briefly, HH st10 chick embryos were harvested from the egg, trimmed of vitelline membrane and extra-embryonic tissue, and washed with PBS (Gibco). Subsequently, whole embryos were placed in a solution of 1 mg/ml dispase for 15 min. and then washed for 5 min. in 0.1 mg/ml DNase1/PBS (Gibco). Using a combination of microsurgery and physical dissociation, the intact neural tube was isolated from the surrounding mesoderm and ectoderm (Fig. 1B). Using the characteristic morphological markers of reiterated constrictions and a tungsten needle, the neural tube was dissection into discrete segments. To maximize the chances to identify transcriptionally regulated events, the neural tube was divided into the major regions of the forebrain (F), midbrain (M) and hindbrain (H). However, within the hindbrain, rhombomere 1 (r1) was dissected on its own in an attempt to elucidate changes in gene transcription unique to the incidence of Fgf8 signaling from the MHB. Tissues were pooled in 1 ml aliquots of Trizol (Gibco) RNA extraction buffer, snap frozen on dry ice and stored at -70°C. To decrease the incidence of false positives in the differential display procedure, sufficient dissections were performed to conduct the entire study in duplicate (series 1: F = 211, M = 200, r1 = 190, hb 214 & series 2: F = 220, M = 207, r1 = 185, hb 187 mRNA pools). To minimize variation in the pooling approach, only st10 (10 somites) embryos were collected. Those embryos with 9, 10 or 11 somites (HH st10-, 10 and 10+ respectively) were fixed in 4% paraformaldehyde for later *in situ *hybridizations.

The use of high quality fully intact mRNA has been established as a critical factor in the representation and reproducibility of mRNA differential display (DD) [43]. To achieve this, we adopted a stringent RNA extraction procedure as detailed in [43]. To check that the integrity of the total RNA had not been compromised by the dissection and proteolytic treatment of the neural tube, it was compared at all stages to total RNA extracted from an untreated/dissected control HH st10 chick embryo (Fig. 1C, lanes 1 vs. 2–5). No difference in the gel profiles was observed for any of the samples, demonstrating that all total RNAs were intact and applicable to differential display RT-PCR. The final RNA concentration was determined spectrophotometrically and stored in aliquots at -70°C.

### Messenger mRNA differential display

Messenger mRNA differential display was performed using the Hieroglyph mRNA Profile System (Genomyx Corporation, Beckman, USA) which is derived from the original procedure described by (37)) [44-46]. Deviations from the original protocol are as described by [42,47,43].

### Reamplification and subcloning

Reproducibly differentially expressed bands in duplicate tissue sample (i.e. series 1 and 2 mRNA pools) were recovered from the dried gel by scoring the outline of the band with a scalpel blade, rehydrating in 2 μl of sterile water then using the blade to transfer the excised gel fragment to a PCR tube containing 6 μl of water. The gel cDNA fragments were reamplified in a 40 μl reaction volume using the same primers and reagent concentrations employed in the original differential display reactions but omitting the radioisotope and using the following cycling parameters: 95°C for 2 min; 30 cycles of 92°C for 15 s, 60°C for 30 s, 72°C for 2 min; 72°C for 10 min. Re-amplified products (10 μl) were visualized on a 1% agarose gel to ensure that a single PCR product of the expected size was obtained (Fig. 1F). The band was purified from the remaining reaction mixture using the Wizard PCR DNA purification system (Promega) and cloned into the pCR-TOPO vector using the TOPO TA cloning system (Invitrogen, U.S.A.) (Fig. 1G). Plasmid DNA was purified using either the Wizard Plus Minipreps kit (Promega) or Qiagen Mini SpinColumn kit (Qiagen).

### Library screening and ExAssist procedures

Chick HH st10 cDNA λZAPII ([48]; Stratagene, USA) clones were plated at a density of ~30,000 plaques/20 cm dish. Approximately 500,000 individual plaque-forming units were screened per clone as per manufacturer's instruction. Plaques that were identified as positive for hybridization were screened until they could be unambiguously isolated (secondary and tertiary screens (Fig. 1I,I' respectively)). Subsequently, pBSSK- plasmid vectors containing the relevant cDNA were excised from the λ phage using the ExAssist protocol as per manufacturer's instructions (Stratagene, USA). Appropriate correspondence between the probe and newly identified plasmid clone was confirmed by both Southern blot (data not shown) and sequence analysis. Where multiple clones were isolated, the longest clone was chosen for full sequencing.

### Construction of chick HH st10 5' RACE library

Due to the lack of highly representative chick cDNA libraries of the appropriate stage and identity and in order to isolate longer cDNA clones from the differential display clones, we decided to construct a neural tube-specific 5' library for Rapid amplification of cDNA ends (RACE). Total RNA (0.2 mg) was collected (as described elsewhere) from 20 HH10 neural tubes that had been purified from adherent mesoderm and ectoderm. Sufficient (500 ng) polyA mRNA was recovered using an oligo dT-magnetic bead kit as described in the manufacturer's instructions (Stratagene, USA) and the 5'RACE library was assembled using the Marathon kit as per manufacturer's guidelines (Clontech, USA).

### Sequencing

Each repeated and re-amplified differentially expressed band was cloned independently at least six times. Clones isolated from the screening of the chick HH st10 cDNA library were sequenced on both strands prior to submission of the sequence to Genbank. Where necessary, the sequence of longer clones was obtained by synthesis of new primers (Oswell, UK) and a walking strategy.

### Single Stranded Conformational Polymorphism (SSCP)

SSCPs were used to determine the complexity of up regulated differential display bands and were performed as described by [42].

### Southern and Northern blot analysis

Southern and Northern blots were performed essentially as described in (49) with modifications as described by (50). Briefly, total RNA was isolated as detailed above from chick HH st10 neural tubes. Following denaturation at 95°C for 10 mins, 10 μg total RNA/blot was loaded and run overnight at constant V (15 V) on a 0.8% (w/v) agarose denaturing-formaldehyde gel [50]. The position of the 28s and 18s rRNA bands were recorded prior to transferring the total RNA to Hybond N+ (Amersham) by capillary blotting. The size of the transcript corresponding to the differential display fragment was determined by hybridization to a ^32^P-labelled probe. Hybridised blots were washed with 0.1% SDS 0.1× SSC at 60°C for 8 hours before being exposed to X ray (Kodak) film overnight at -70°C, The size of the resultant hybridized bands were calculated by comparing their relative location to the 28s and 18s rRNA transcripts.

### Gene identities and bioinformatics

The identities of the isolated cDNA fragments or their corresponding library clone were revealed by either BLASTN or BLASTX searches of the sequences submitted in either BBSRC/UMIST chicken EST repository (84) or the non-redundant Genbank database (85). Where appropriate the identity of a cDNA sequence was inferred from a near identical match to an ortholog (e.g. chick to human). To give further insight into potential function, various other sequence databases were queried. For example, using the NCBI Conserved Domain Search (86) to reveal the presence of well-conserved functional motifs.

### Whole mount *in situ *hybridization

Whole mount *in situ *hybridisation with digoxigenin and fluorescein-labelled riboprobes was performed as described by Wilkinson (1992) with the exception that the embryos were fixed in MEMFA (100 mM Mops, 2 mM EGTA, 1 mM MgSO4, 3.7% (v/v) formaldehyde). Differential display cDNAs cloned into the pCR-TOPO vector of those isolated from library screening (pBS SK- (Stratagene, USA)) were used as templates for synthesising DIG-labelled riborprobes. Probe integrity and approximate concentration was checked by agarose gel electophoresis and approximately 1 μg DIG labelled RNA/ml hybridisation buffer was used with at least 3 HH st9-11 embryos. Where poor *in situ *signal was obtained, and where possible, the equivalent chick cDNA plasmid was obtained from the MRC Geneservice (Cambridge, UK). The identity of all plasmids was checked by DNA sequence analysis prior to probe generation. Embryo sectioning was performed as described by (18).

## Authors' contributions

David Chambers assisted in the design and execution the work described herein and Ivor Mason designed, supervised and advised on the project throughout.

## References

[B1] Kiecker C, Lumsden A (2005). Compartments and their boundaries in vertebrate brain development. Nat Rev Neurosci.

[B2] Figdor MD, Stern CD (1993). Segmental origin of embryonic diencephalons. Nature.

[B3] Larsen CW, Zeltser L, Lumsden A (2001). Boundary formation and compartition in the avian diencephalon. J Neurosci.

[B4] Zeltser L, Larsen CW, Lumsden A (2001). A new developmental compartment in the forebrain regulated by lunatic fringe. Nat Neurosci.

[B5] Kiecker C, Lumsden A (2004). Hedgehog signaling from the ZLI regulated diencephalic regional identity. Nat Neurosci.

[B6] Simeone A (2000). Positioning the isthmic organizer where *Otx*2 and *Gbx*2 meet. Trends Genet.

[B7] Liu A, Joyner AL (2001). Early anterior/posterior patterning of the midbrain and cerebellum. Annu Rev Neurosci.

[B8] Wurst W, Bally-Cuif L (2001). Neural plate patterning: upstream and downstream of the isthmic organizer. Nat Rev Neurosci.

[B9] Heikinheimo M, Lawshe A, Shackleford GM, Wilson DB, MacArthur CA (1994). Fgf-8 expression in the post-gastrulation mouse suggests roles in the development of the face, limbs and central nervous system. Mech Dev.

[B10] Ohuchi H, Yoshioka H, Tanaka A, Kawakami Y, Nohno T, Noji S (1994). Involvement of androgen-induced growth factor (FGF-8) gene in mouse embryogenesis and morphogenesis. Biochem Biophys Res Commun.

[B11] Crossley PH, Martin GR (1995). The mouse Fgf8 gene encodes a family of polypeptides and is expressed in regions that direct outgrowth andpatterning in the developing embryo. Development.

[B12] Mahmood R, Bresnick J, Hornbruch A, Mahony K, Morton N, Colquhoun K, Martin P, Lumsden A, Dickson C, Mason I (1995). FGF-8 in the mouse embryo: a role in the initiation and maintenance of limb bud outgrowth. Curr Biol.

[B13] Bueno D, Skinner J, Abud H, Heath JK (1996). Spatial and temporal relationships between Shh, Fgf4, and Fgf8 gene expression at diverse signalling centers during mouse development. Dev Dyn.

[B14] Crossley PH, Martinez S, Martin GR (1996). Midbrain development induced by FGF8 in the chick embryo. Nature.

[B15] Vogel A, Rodriguez C, Izpisua-Belmonte JC (1996). Involvement of FGF-8 in initiation, outgrowth and patterning of the vertebrate limb. Development.

[B16] Christen B, Slack JM (1997). FGF-8 is associated with anteroposterior patterning and limb regeneration in Xenopus. Dev Biol.

[B17] Riefers F, Bohli H, Walsh E, Crossley P, Stanier D, Brand M (1998). *Fgf8 *is mutated in zebrafish *acerebellar *(*ace*) mutants and is required for maintenance of midbrain-hindbrain boundary development and somitogenesis. Development.

[B18] Shamim H, Mahmood R, Logan C, Doherty P, Lumsden A, Mason I (1999). Sequential roles for Fgf4, En1 and Fgf8 in specification and regionalisation of the midbrain. Development.

[B19] Sheikh H, Mason I (1996). Polarising activity of FGF-8 in the avian midbrain. Int J Dev Biol.

[B20] Martinez S, Crossley PH, Cobos I, Rubenstein JL, Martin GR (1999). FGF8 induces formation of an ectopic isthmic organizer and isthmocerebellar development via a repressive effect on Otx2 expression. Development.

[B21] Prakash N, Wurst W (2004). Specification of the midbrain territory. Cell Tissue Res.

[B22] Lumsden A, Keynes R (1989). Segmental patterns of neuronal development in the chick hindbrain. Nature.

[B23] Lumsden A (2004). Segmentation and compartition in the early avian hindbrain. Mech Dev.

[B24] Wingate R, Hatten ME (1999). Cerebellar rhombic lip derivatives. Development.

[B25] Dupe V, Lumsden A (2001). Hindbrain patterning involves graded responses to retinoic acid signalling. Development.

[B26] Lumsden A, Krumlauf R (1996). Patterning the vertebrate neuraxis. Science.

[B27] Maden M (2002). Retinoid signalling in the development of the central nervous system. Nature Neurosci Reviews.

[B28] Krumlauf R (1994). Hox genes in vertebrate development. Cell.

[B29] Studer M, Lumsden A, Ariza-McNaughton L, Bradley A, Krumlauf R (1996). Altered segmental identity and abnormal migration of motor neurons in mice lacking *Hoxb*1. Nature.

[B30] Bell E, Wingate R, Lumsden A (1999). Homeotic transformation of rhombomere identity after localized *Hoxb*1 misexpression. Science.

[B31] Manzanares M, Cordes S, Ariza-McNaughton L, Sadl V, Maruthainar K, Barsh G, Krumlauf R (1999). Conserved and distinct roles of kreisler in regulation of the paralogous *Hoxa*3 and *Hoxb*3 genes. Development.

[B32] Manzanares M, Trainor P, Nonchev S, Ariza-McNaughton L, Brodie J, Gould A, Marshall H, Morrison A, Kwan CT, Sham MH, Wilkinson DG, Krumlauf R (1999). The role of kreisler in segmentation during hindbrain development. Dev Biol.

[B33] Sham M, Vesque C, Nonchev S, Marshall H, Frain M, Das Gupta R (1993). The zinc finger gene Krox-20 regulates Hox-2.8 (*Hoxb*2) during hindbrain segmentation. Cell.

[B34] Nonchev S, Vesque C, Maconochie M, Seitanidou T, Ariza-MacNaughton L, Frain M, Marshall H, Sham MH, Krumlauf R, Charnay P (1996). Segmental expression of *Hoxa*2 in the hindbrain is directly regulated by Krox-20. Development.

[B35] Giudicelli F, Taillebourg E, Charnay P, Gilardi-Hebenstreit P (2001). Krox-20 patterns the hindbrain through both cell-autonomous and non cell-autonomous mechanisms. Genes Dev.

[B36] Christiansen JH, Coles EG, Robinson V, Pasini A, Wilkinson DG (2001). Screening from a subtracted embryonic chick hindbrain cDNA library: identification of genes expressed during hindbrain, midbrain and cranial neural crest development. Mech Dev.

[B37] Liang P, Pardee AB (1992). Differential display of eukaryotic messenger RNA by means of the polymerase chain reaction. Science.

[B38] Leslie RA, Robertson HA, Eds (2000). Differential Display: A Practical Approach.

[B39] Hogenesch JB, Ching KA, Batalov S, Su AI, Walker JR, Zhou Y, Kay SA, Schult PG, Cooke MP (2001). A comparison of the Celera and Ensembl predicted gene sets reveals little overlap in novel genes. Cell.

[B40] Saha S, Sparks AB, Rago C, Akmaev V, Wang CJ, Vogelstein B, Kinzler KW, Velculescu VE (2002). Using the transcriptome to annotate the genome. Nat Biotechnol.

[B41] Hamburger V, Hamilton HL (1951). A series of normal stages in the development of the chick embryo. J Morphol.

[B42] Chambers D, Medhurst AD, Walsh FS, Price J, Mason I (2000). Differential display of genes expressed at the midbrain – hindbrain junction identifies sprouty2: an FGF8-inducible member of a family of intracellular FGF antagonists. Mol Cell Neurosci.

[B43] Medhurst AD, Chambers D, Gray J, Davis JB, Mason I, Jenner P, Newton R, Leslie RA, Robertson HA (2000). Practical aspects of the experimental design for differential display of transcripts obtained from complex tissues. Differential Display: A Practical Approach.

[B44] Bauer D, Muller H, Reich J, Riedel H, Ahrenkiel V, Warthoe P, Strauss M (1993). Identification of differentially expressed mRNA species by an improved display technique (DDRT-PCR). Nucl Acids Res.

[B45] Averboukh L, Douglas SA, Zhao S, Lowe K, Maher J, Pardee AB (1996). Better gel resolution and longer cDNAs increase the precision of differential display. BioTechniques.

[B46] Medhurst AD, Chambers D, Gray J, Davis JB, Mason I, Jenner P, Newton R, Leslie RA, Robertson HA (2000). Practical aspects of the experimental design for differential display of transcripts obtained from complex tissues. Differential Display: A Practical Approach.

[B47] Newton RA, Bingham S, Davey PD, Medhurst AD, Piercy V, Raval P, Parsons AA, Sanger GJ, Case CP, Lawson SN (2000). Identification of differentially expressed genes in dorsal root ganglia following partial sciatic nerve injury. Neuroscience.

[B48] Nieto MA, Sargent MG, Wilkinson DG, Cooke J (1994). Control of cell behavior during vertebrate development by Slug, a zinc finger gene. Science.

[B49] Costigan M, Mannion RJ, Kendall G, Lewis SE, Campagna JA, Coggeshall RE, Meridith-Middleton J, Tate S, Woolf CJ (1998). Heat shock protein 27: developmental regulation and expression after peripheral nerve injury. J Neuroscience.

[B50] Sambrook J, Fritsch T, Maniatis T (1989). Molecular Cloning: A Laboratory Manual.

[B51] Sheets MD, Ogg SC, Wickens MP (1990). Point mutations in AAUAAA and the poly (A) addition site: effects on the accuracy and efficiency of cleavage and polyadenylation *in vitro*. Nucl Acids Res.

[B52] Chambers D (2002). Chick gene frenzy. Trends Genet.

[B53] Wurst W, Auerbach AB, Joyner AL (1994). Multiple developmental defects in Engrailed-1 mutant mice: an early mid- hindbrain deletion and patterning defects in forelimbs and sternum. Development.

[B54] Frohman MA, Martin GR, Cordes SP, Halamek LP, Barsh GS (1993). Altered rhombomere-specific gene expression and hyoid bone differentiation in the mouse segmentation mutant, *kreisler *(*kr*). Development.

[B55] McKay I, Muchamore I, Krumaluf R, Maden M, Lumsden A, Lewis J The kreisler mouse: a hindbrain segmentation mutant that lacks two rhombomeres. Development.

[B56] Walshe J, Mason I (2000). Expression of FGFR1, FGFR2 and FGFR3 during early neural development in the chick embryo. Mech Dev.

[B57] Kim HJ, Sagi DB (2004). Modulation of signalling by sprouty: a developing story. Nat Neurosci.

[B58] Basson MA, Akbulut S, Watson-Johnson J, Simon R, Carroll TJ, Shakya R, Gross I, Martin GR, Lufkin T, McMahon AP, Wilson PD, Costantini FD, Mason IJ, Licht JD (2005). Sprouty1 is a critical regulator of GDNF/RET-mediated kidney induction. Dev Cell.

[B59] Puelles L, Rubenstein JL (2003). Forebrain gene expression domains and the evolving prosomeric model. Trends Neurosci.

[B60] Brekken RA, Sage EH (2000). a matricellular protein: at the crossroads of cell-matrix. Matrix Biol.

[B61] Colognato H, Yurchenco PD (2000). Form and function: the laminin family of heterotrimers. Dev Dyn.

[B62] Pfeffer PL, Payer B, Reim G, Pasca di Magliano M, Busslinger M (2002). The activation and maintenance of *Pax2 *expression at the mid-hindbrain boundary is controlled by separate enhancers. Development.

[B63] Merenmies J, Rauvala H (1990). Molecular cloning of the 18-kDa growth-associated protein of developing brain. J Bio Chem.

[B64] Rauvala H (1989). An 18-kd heparin-binding protein of developing brain that is distinct from fibroblast growth factors. EMBO J.

[B65] Kilpelainen I, Kaksonen M, Avikainen H, Fath M, Linhardt RJ, Raulo E, Rauvala H (2000). Heparin-binding growth-associated molecule contains two heparin-binding beta-sheet domains that are homologous to the thrombospondin type I repeat. J Biol Chem.

[B66] Matsubara S, Tomomura M, Kadomatsu K, Muramatsu T (1990). Structure of a retinoic acid-responsive gene, MK, which is transiently activated during the differentiation of embryonal carcinoma cells and the mid-gestation period of mouse embryogenesis. J Biol Chem.

[B67] Hienola A, Pekkanen M, Raulo E, Vanttola P, Rauvala H (2004). HB-GAM inhibits proliferation and enhances differentiation of neural stem cells. Mol Cell Neurosci.

[B68] Ornitz DM, Itoh N (2001). Fibroblast growth factors. Genome Biol.

[B69] Dubrulle J, Pourquie O (2004). Coupling segmentation to axis formation. Development.

[B70] Gebauer F, Hentze MW (2004). Molecular mechanisms of translational control. Nature Reviews.

[B71] Chambers D, I Mason (2000). Expression of *sprouty2 *during early development of the chick embryo is coincident with known sites of FGF signalling. Mech Dev.

[B72] Stoker A, Dutta R (1998). Protein tyrosine phosphatases and neural development. Bioessays.

[B73] Ensslen-Craig SE, Brady-Kalnay SM (2004). Receptor protein tyrosine phosphatases regulate neural development and axon guidance. Dev Biol.

[B74] Kokel M, Borland CZ, DeLong L, Horvitz HR, Stem MJ (1998). *clr-I *encodes a receptor tyrosine phosphatase that negatively regulates an FGF receptor signaling pathway in Caenorhabditis elegans. Genes Dev.

[B75] Kawakami Y, Rodriguez-Leon J, Koth CM, Buscher D, Itoh T, Raya A, Ng JK, Esteban CR, Takahashi S, Henrique D, Schwarz MF, Asahara H, Izpisua Belmonte JC (2003). MKP3 mediates the cellular response to FGF8 signalling in the vertebrate limb. Nature Cell Biol.

[B76] Gustafson AL, Mason I (2000). Expression of receptor tyrosine phosphatase gamma during early development of the chick embryo. Mech Dev.

[B77] Maroon H, Walshe J, Mahmood R, Kiefer P, Dickson C, Mason I (2002). Fgf3 and Fgf8 are required together for formation of the otic placode. Development.

[B78] Schmid RS, Maness PF, Kalverboer AF, Gramsbergen A (2001). Cell recognition molecules and disorders of neurodevelopment. Handbook on brain and behavior in human development.

[B79] Schmid RS, Pruitt WM, Maness PF (2000). An MAPK signaling pathway mediates neurite outgrowth on L1 and requires Src-dependent endocytosis. J Neurosci.

[B80] Demyanenko GP, Maness PF (2002). The L1 Cell Adhesion Molecule Is Essential for Topographic Mapping of Retinal Axons. J Neuroscience.

[B81] Wilson L, Gale E, Chambers D, Maden M (2004). Retinoic acid and the control of dorsoventral patterning in the avian spinal cord. Dev Biol.

[B82] National Center for Biotechnology Information Genome BLAST Server. http://www.ncbi.nlm.nih.gov/BLAST/genomes.

[B83] UTResource. http://bighost.area.ba.cnr.it/BIG/UTRHome.

[B84] BBSRC Chick EST Database. http://www.chick.umist.ac.uk.

[B85] National Center for Biotechnology Information BLAST Server. http://www.ncbi.nlm.nih.gov/BLAST/.

[B86] National Center for Biotechnology Information Conserved Domain Search. http://www.ncbi.nlm.nih.gov/Structure/cdd/wrpsb.cgi.

